# Exploring psychological resilience of entrepreneurial college students for post-pandemic pedagogy: The mediating role of self-efficacy

**DOI:** 10.3389/fpsyg.2022.1001110

**Published:** 2022-09-08

**Authors:** Hui Guo, Yu Zhang, Yaqi Tian, Wenxiu Zheng, Limeng Ying

**Affiliations:** ^1^School of Management, Wenzhou Business College, Wenzhou, China; ^2^Department of Public and International Affairs, City University of Hong Kong, Hong Kong, Hong Kong SAR, China; ^3^Department of Diplomacy and Foreign Affairs Management, China Foreign Affairs University, Beijing, China; ^4^School of Digital Economy and Trade, Wenzhou Polytechnic, Wenzhou, China

**Keywords:** psychological resilience, pedagogy, self-efficacy, entrepreneurial college students, SEM

## Abstract

The psychological impact of the COVID-19 epidemic on college students is an important topic. With the entry of the post-epidemic era, how universities can better improve students’ psychological resilience in teaching is the research topic of this article. In the form of a questionnaire survey, some entrepreneurial college students investigated the loneliness and psychological resilience of college students after the outbreak of the epidemic and explored the role of self-efficacy in it. The data is collected online through cooperation with an entrepreneurial event, and the participating students are asked for background information such as colleges, grade, and majors. After collecting this information, they answered a series of simplified scale questions about loneliness, self-efficacy, and psychological resilience. In the end, a total of 200 questionnaires from different universities were collected, and the structural equation model was used to explore the role of self-efficacy. The results show that: loneliness has a significant negative effect on Self-efficacy, β = -0.292, *p* < 0.001; Self-efficacy has a significant positive effect on psychological resilience, β = 0.556, *p* < 0.0 01; loneliness has a significant negative effect on psychological resilience, β = -0.244, *p* < 0.01. Self-Efficacy has a significant intermediate effect in loneliness and psychological resilience, with an effective value of -0.111 and *p* < 0.01. The results show that this exploratory survey finds it important to provide targeted personal self-efficacy improvement activities for college students with a strong sense of loneliness and to combine school and family education organically to help college students form a healthy and upward mentality to better cope with the unknown and troubles caused by the epidemic, which will help improve the psychological resilience of college students in the epidemic.

## Introduction

At the beginning of 2020, with the outbreak of COVID-19, the daily life of people around the world changed a lot. In response to the COVID-19 epidemic, governments took several administrative measures, such as social distancing, home quarantine, and travel restrictions, to reduce the spread of COVID-19. College students were almost limited to their homes. Sharma et al. (2020) found that individuals during epidemic isolation will have negative and repressed emotions, which have reduced people’s life satisfaction. In recent years, with the epidemic situation being better and the gradual relaxation of epidemic prevention policies in China, college students have also begun to return to school, but excessive psychological pressure, anxiety, and other problems remain in some of them.

Due to long-term home isolation, loneliness has been an important factor in describing the mental health background of college students and a reflection of a closed heart. Perlman et al. (1984) defines loneliness as an individual’s feelings about the quality and quantity of social relations and it is generated when an individual’s actual social relationship can’t meet their expectations. Qualter et al. (2010) believes that loneliness is a negative emotional experience, which comes from dissatisfaction with the individual’s social network, and it is an important factor affecting mental health and will also have an impact on the development of psychological function. Based on the concept of loneliness at home and abroad, Mellor et al. (2008) puts forward that loneliness is a negative subjective emotional experience when an individual’s interpersonal relationship does not meet his satisfaction. This experience usually occurs when there is a gap between his actual communication level and his expected communication level, accompanied by feelings such as loneliness and helplessness. In general, temporary or accidental loneliness will not cause psychological behavioral disorders, but long-term or severe loneliness can cause certain emotional disorders and reduce people’s mental health. Loneliness will also increase the isolation and alienation from others and society, and the isolation and alienation will then strengthen people’s sense of loneliness, which will inevitably lead to personality disorders of isolated individuals over time.

In addition to the objective growth environment (family and society), the factors affecting loneliness mainly include the habit of self-evaluation, communicative ability, and specific emotions. Among these psychological problems, the focus of this paper is whether loneliness has an impact on psychological resilience. Psychological resilience, also known as mental resilience, refers to “an individual’s behavioral tendency to change itself to adapt to changing environments and the ability to recover from stressful situations” (Block and Block, 1980), and resilience is a psychological trait possessed by individuals and a process of interaction between individuals and the environment (Bolton et al., 2017). Psychological resilience is a manifestation of self-healing power. An important therapeutic hypothesis in psychotherapy is that the client has self-healing power and can self-heal the trauma he has suffered (Bohart, 2000). College students are a special group. They are in the transition stage from adolescence to adulthood. Their psychological function is still in an unstable stage, and their self-regulation and anti-pressure abilities are poor. In the face of pressure and stress, it is very easy for them to have a variety of psychological problems. This paper assumes that loneliness is related to psychological resilience because some literature has demonstrated a similar association. Dong and Zhang (2013) found in their research on left-behind children in Yunnan minority areas that the better their psychological resilience is, the weaker their loneliness is, and the weaker their psychological resilience, the stronger their loneliness is. Among them, emotional control has a great influence on psychological resilience. In addition, positive cognition and family support also have a significant impact on the psychological resilience of migrant children (Ye et al., 2016). Research by Tang et al. (2021) on the structural model of adolescent psychological resilience found that if a teenager has a high level of psychological resilience, then the level of loneliness he feels in his study and life is very low, the level of loneliness experienced by those teenagers with medium psychological resilience is also at the medium level, while the level of loneliness with the lowest level of psychological resilience is the highest. Wang F. et al. (2019) studied children’s interpersonal relationships and found that children with better psychological resilience also have higher cognitive scores on communicating with others, while children with poor psychological resilience have lower cognitive scores on communicating with others, and the good or bad of interpersonal relationships have significant predictive values for individual loneliness. According to Kaloeti et al. (2019), college students with high scores of psychological resilience can better adapt to school life, so that it is easier for them to establish good interpersonal relationships and they’re less likely to feel lonely.

In addition, the role of self-efficacy is worth discussing. Because loneliness is largely endogenous, it is difficult to change through external forces. Therefore, this paper puts forward the hypothesis that the negative relationship between loneliness and psychological resilience will be regulated by personal self-efficacy. The concept of self-efficacy was first proposed by the American psychologist Bandura et al. (1999), who explained it as an individual’s belief and assessment of their ability when achieving certain expected goals. In the past studies, it has been confirmed that the influencing factors of self-efficacy include concepts, attitudes, consciousness, methods, and parents’ educational methods, etc. In addition, the interpersonal relationship among social factors also has an important impact on self-efficacy. Bing et al. (2022) confirmed that emotional regulation self-efficacy has a significant predictive effect on academic achievements. Buriæ and Macuka (2018) shows that there is a significant correlation between teachers’ emotions, self-efficacy, and work commitment. Bandura et al. (1999) and his followers believe that self-efficacy is an individual’s belief in and assessment of his or her ability when achieving certain expected goals. After conducting a questionnaire survey on enterprise employees, Joie-La Marle et al. (2021) found that the general sense of self-efficacy has a significant positive predictive effect on adaptive performance. To sum up, this paper believes that if the role of self-efficacy can be successfully detected, it will enable schools to provide targeted help to students with a strong sense of loneliness in the post-epidemic era, which has important reference value.

In recent years, some scholars have carried out research on the psychological resilience of college student “entrepreneurs.” Ke (2009) believes psychological capital is mainly composed of transactional psychological capital and relational psychological capital, each of which includes four parts. Bockorny and Youssef-Morgan (2019) believe courage in psychological capital is as important as hope and optimism and determines life satisfaction and entrepreneurial ability. Based on a meta-analysis of psychological capital, it is proposed that self-confidence, willingness to achieve, and entrepreneurial orientation have positive impacts on entrepreneurship (Frese and Gielnik, 2014). However, researches on psychological resilience among entrepreneurial college students are still lacking. Since the participants are entrepreneurial college students, therefore, this paper investigated their loneliness and psychological resilience with a questionnaire survey. This study will understand entrepreneurial college students’ psychological condition in response to stress and analyze the relationship among loneliness, self-efficacy, and psychological resilience, which can provide a reference for promoting the mental health development of college students. This study attempts to fill the gap of research on the psychological status of post- epidemic college students and is conducive to providing a reference for the analysis of the epidemic based on the research of college students in this paper. And from the perspective of psychology, this study has taken measures to improve the psychological resilience of post-epidemic college students and formulated corresponding intervention strategies.

## Methods

### Procedure and participants

The research was conducted in June 2022, and the research objects are participants of an entrepreneurial competition in Wenzhou, Zhejiang province, who were all students in colleges and universities in Zhejiang province. Questionnaires were distributed to each group of participants *via* email. The reason why participants in the entrepreneurial competition were selected as the research objects of this study is that entrepreneurs have special psychological qualities and the internal personality differences of different entrepreneurs lead to different entrepreneurial results. Entrepreneurs’ resources, personality characteristics, and psychological quality determine the differences in entrepreneurship. Entrepreneurs’ business ideals, dedication, and perseverance are of great importance (Kets de Vries, 1985). Students with an entrepreneurial spirit tend to have greater variation in self-efficacy, which is more helpful for us to explore differences at the statistical level. Questionnaires were distributed to 205 students by stratified random sampling, and 200 valid questionnaires were retrieved, with an effective response rate of 97.78%. Among the objects: 72 are men (36.00%) and 128 are women (64.00%); 67 families live in rural areas (33.50%) and 53 (26.50%) in urban areas. In China, there is a conventional classification of the level of colleges and universities. And we classified the colleges and universities according to the general cognition of the society and obtained participants from different levels of colleges and universities. Among them, 63 (31.50%) are from 211/985/ double first-class universities, 62 (31.00%) from first-class universities, 52 (26.00%) from second-class universities (including private undergraduate universities), and 23 (11.50%) from junior colleges. In terms of grade, in our samples, 67 cases are freshmen (33.50%), 72 sophomores (36.00%), 54 juniors (27.00%), and 7 (3.50%) seniors. These 200 people were classified by discipline, including 30 majoring in Medical (15.00%), 52 in Science (26.00%), 48 in Social Science (24.00%), and 22 in Others (11.00%). In terms of age, the minimum age is 18, the maximum age is 25, the average age is 20.29, and the standard deviation is 1.4223.

### Measurements

#### Loneness

Researchers represented by Daniel Russell in University of California, Los Angeles (UCLA) developed the UCLA Loneliness Scale which is widely applicable to understand the lonliness in a wide range of groups and has high reliability and validity. The α coefficient of the scale is 0.87, which can measure loneliness caused by the gap between the expectation of social interaction and the experience. Considering that this paper aims at the discussion related to Post-Pandemic Pedagogy, the original scale was reduced by this article and finally, five items were retained. For all these items, the respondents were asked to show how much they agreed with the statements on a 5-point scale, from one (no, I entirely disagree) to five (yes, I entirely agree). Thus, a higher score in structure reflects a higher level of measured characteristics. Specific items are as follows:

Loneliness 1: I always feel that no friends will help me.
Loneliness 2: I always feel neglected.
Loneliness 3: I always feel alienated from others.
Loneliness 4: I always feel sad because I seldom associate with people.
Loneliness 5: Although there are people around me, I always feel that no one cares about me.

#### Self-efficacy

Self-efficacy is to evaluate whether you have the confidence to deal with all kinds of pressures in life. Self-efficacy is the core concept in Bandura’s social cognitive theory (Beauchamp et al., 2019; Chen and Wu, 2021), which is different from outcome expectation. The outcome expectation refers to an individual’s perception of the consequences of his or her actions, while self-efficacy refers to the control or dominating of his or her actions. A person who believes that he or she can handle all kinds of things will be more active and proactive in life. This perception of “can do” reflects a sense of control over the environment, so the sense of self-efficacy reflects a belief that an individual can take appropriate actions to face environmental challenges. Self-efficacy looks at an individual’s ability to deal with all kinds of stress in life from a confident perspective. The Chinese version of the General Self-efficacy Scale (GSES) was translated and revised by Wang Caikang. In the revised Chinese version of the scale, the internal consistency coefficient and retest reliability are, respectively, 0.87 and 0.83, with good predictive validity (Wang N. et al., 2019). As a pilot study, this scale has been simplified in this paper, and to better meet the requirements of modeling, a five-point system has been used for Loneliness measurements.

Self-efficacy 1: If I try my best, I can always solve the problem.
Self-efficacy 2: It’s easy for me to stick to my ideals and achieve goals.
Self-efficacy 3: I am confident that I can successfully deal with any emergencies.
Self-efficacy 4: With my intelligence, I can cope with unexpected situations.
Self-efficacy 5: I can face difficulties calmly because I believe in my ability.
Self-efficacy 6: No matter what happens to me, I can deal with it freely.

#### Psychological resilience

Psychological resilience (Psycho Resi) is the state of the subject’s psychological and behavioral response to the changing environment. This state is dynamic. It changes with the change of the environment and achieves dynamic regulation and adaptation to the environment. As early as in the 1990s, Chinese workers received foreign scholars to measure the level of psychological resilience through the psychological resilience scale. Since then, this concept has always been a frequently discussed topic, which has also made the psychological resilience scale widely used. Among them, the scale on the psychological resilience of college students is the revised version of Yu and Zhang (2007) of the Chinese University of Hong Kong, which has good reliability and validity. The scale consists of three scales of resilience, strength, and optimism, with a total of 25 items, using the 5-point scale evaluation method. However, to make a preliminary exploration in this paper, we have simplified the scale into the following items and adopted a five-point system:

Psycho Resi 1: Sometimes I encounter problems that can’t be easily solved, and the god of luck will bless me.
Psycho Resi 2: When facing problems, I try to treat them with a positive attitude.
Psycho Resi 3: I will believe that the tempering I have experienced will make me stronger.
Psycho Resi 4: Even if I experience illness or suffering, I can recover.
Psycho Resi 5: I won’t be easily knocked down by suffering.
Psycho Resi 6: I think I am a strong person.
Psycho Resi 7: I can control my negative emotions.

### Analysis

The Structural Equation Model (SEM) is a verifiable multivariate statistical method, which is now widely used in academic research in various fields to explain the relationship between variables through variable covariance matrices (Chen and Zhang, 2021). It integrates influencing factor analysis and related path analysis and can be used to process and analyze complex multivariate research data. SEM models can not only process and analyze complex data but also estimate its latent variables and measure complex variables. At the same time, the model allows measurement errors between independent variables and dependent variables. By establishing, estimating, and testing causal relations, the SEM model has become a common and important statistical method. Since the traditional regression does not consider the relationship between submerged variables and the structural equation model analysis considers the common variation among all latent variables, this paper decides to use the structural equation model to further verify the assumptions we put forward.

## Results

### Credit analysis and exploratory factor analysis

Descriptive statistics are carried out on all continuous variables in this study, including mean value, standard deviation, skewness, and kurtosis. Kline (2015) believes that if the absolute value of the sample’s observed variable is greater than 3 and the absolute peak value is greater than 10, the data may deviate from the normal score. The descriptive statistical results of the questions can be found in the Appendix, and the results show that the absolute skewness value of all questions in this study is less than 3 and the absolute kurtosis value is less than 10. The sample data obey the normal distribution and is suitable for subsequent analysis.

The reliability test, also known as reliability analysis, measures the credibility of the questionnaire (Zhang et al., 2022). It mainly determines whether the questionnaire data is stable and reliable, and generally analyzes the credibility of the questionnaire based on the Cronbach Alpha (α) value. The Cronbach Alpha (α) value is at 0–1, and the closer its value is to 1, the higher the reliability of the questionnaire data is. If the α of the measurement dimension is higher than 0.8, the internal consistency of the measurement dimension is considered to be high; the values of loneliness, SelfEfficacy, and PsychoResi’s Cronbach’s Alpha (α) are 0.898 and 0.881 and 0.833, respectively. This shows that the stability of the results is high, and the reliable quality of the scale is very good.

To test the quality of the questions in the questionnaire, it is necessary to carry out an Exploratory Factor Analysis (EFA) of dimension questions. Exploratory factor analysis requires that the data of KMO (Kaiser-Meyer-Olkin Measure of Sampling Adequacy) is greater than 0.6, the Bartlett Spherical Test is significant, and the factor load should be as large as possible after factor analysis. There is a large crossroad, and the cumulative variance interpretation rate should be greater than 40% (Hoyt and Kerns, 1999). The results show that the KMO value of the scaling topic is 0.853 and the Bartlett Spherical Test is significant (*p* < 0.001). The data are suitable for exploratory factor analysis.

The eigenvalue analysis shows that there are six components with eigenvalues greater than 1, which cumulatively explains 69.283% of the variance. Six components are extracted and rotated by using the maximum variance method. The results show that the lowest factor load is greater than 0.5 and there is no large cross loading. Except for PsychoResi1 and PsychoResi7, the structure between the factors is clear and consistent with expectations. It has good structural validity.

### Verification factor analysis

The validity evaluation of the scale mainly includes content validity and structural validity. The evaluation indicators selected in this study are adapted from mature scales at home and abroad and formed under repeated refinement by relevant experts and teachers. Exploratory factor analysis is also ideal, which can indicate that the content validity is better; structural validity can be tested by verification factor analysis, including convergence and distinguishing validity. Observations of convergence validity include factor load, combined reliability (CR), and average extraction variance (AVE).

In the verification factor analysis, the factor load of the question should be higher than 0.6, and the fit of the model should meet the corresponding standard. If CR > 0.7 and AVE > 0.5, based on the Composite Reliability (CR) and the Average Variance Extracted (AVE) of standardized factor load calculation dimensions, the measurement dimension will have a good Convergent Validity (Hair, 2011).

To determine that each variable in the study represents its different configurations, this study uses AMOS software to analyze the validity of all variables in the model and calculates the fitting parameters of the model. In this study, the model fitting degree can be verified by χ2/df, GFI, RMSEA, CFI, IFI, TLI, PGFI, and PNFI. χ2/df generally ranges between 1 and 3, and the smaller this value is, the better the fitness of the model is, in addition, the larger the TLI and CFI are, the better and the maximum should not be greater than 1, and the RMSEA should be less than 0.08 and the smaller it becomes, the better. As can be seen from Table 1, except for GFI (acceptability), multiple fitting indicators in the model have reached the ideal situation, and the fitting effect is the best. Therefore, all variables in this study have good distinguishing validity and can represent their respective dimensions. Except for PsychoResi 1, the measure term factor load of each potential variable is between 0.50 and 0.8, which is greater than the 0.50 theoretical value, and it is significant at the level of *p* < 0.001. Most CR values are greater than 0.70, which are much higher than 0.60. It can be seen that all these three observation indicators have met the basic requirements of the test, indicating that the survey scale has a good convergence effect.

**TABLE 1:** Verification factor analysis results.

Names of indicators		Evaluation criteria	Numbers
Absolute adaptation index	CMIN/DF	<3	1.898
	GFI	>0.9	0.842
	RMSEA	<0.08	0.067
Relative adaptation index	CFI	>0.9	0.907
	IFI	>0.9	0.908
Simple adaptation index	TLI	>0.9	0.895
	PGFI	>0.5	0.686
	PNFI	>0.5	0.728

### Correlation analysis

Correlation analysis can be used to verify whether there is a correlation between variables, as well as the direction and extent of the correlation. In this study, Pearson correlation analysis was used to analyze control variables and independent variables. The average score of each dimension question is divided as a dimension score, and the Pearson correlation analysis of each dimension is carried out as follows: loneliness (M = 2.250, SD = 0.947), SelfEfficacy (M = 3.195, SD = 0.808), PsychoResi (M = 3.714, SD = 0.620). The correlation coefficient results are: loneliness-SelfEfficacy (-0.244**), loneliness-SelfEfficacy (-0.368**), SelfEfficacy- PsychoResi (0.602**).

Loneliness can be seen to have a significant negative correlation with SelfEfficacy and PsychoResi; SelfEfficacy has a significant positive correlation with PsychoResi. The results of the above correlation analysis only preliminarily verify the correlation between the variables in this study, so the subsequent hypothesis test needs to be carried out through further analysis.

### Model evaluation

The goodness-of-fit of a model is an indicator used to evaluate the degree of matching between the theoretical model and the actual data. Usually, the higher the goodness-of-fit of the model is, the better the practicality of the model is. To investigate the fitting degree of a model, a series of indicators are usually selected to comprehensively evaluate the model after the parameter estimation is completed. The specific evaluation indicators and their categories are shown in Table 2. We compare the evaluation criteria of each indicator with the actual fitting value of the model to test the fitting effect of the model.

**TABLE 2:** Fit test form of the model.

Names of indicators		Evaluation criteria	Numbers	Pass the inspection or not
Absolute adaptation index	CMIN/DF	<3	1.670	Ideal
	GFI	>0.9	0.918	Acceptable
	RMSEA	<0.08	0.058	Ideal
Relative adaptation index	CFI	>0.9	0.964	Ideal
	IFI	>0.9	0.916	Ideal
Simple adaptation index	NFI	>0.9	0.964	Acceptable
	PGFI	>0.5	0.666	Ideal
	PNFI	>0.5	0.759	Ideal

The structural equation model has a fitting index, of which the ideal CMIN/DF value should be less than 5, and the strict standard should be less than 3; the CFI, IFI, GFI, TLI, and NFI which are greater than 0.9 are good, and if these values are practically greater than 0.8, they also can be acceptable; RMSEA should be less than 0.10, and it is good when the value is less than 0.08. The values of the adaptation indicators of this model are as follows: CMIN/DF = 1.670, CFI = 0.964, IFI = 0.916, NFI = 0.964, RMSEA = 0.05. Judging from the existing results, all these fitting indexes have passed the test, indicating that the overall fit of the structural equation model is good, has a certain application value, and can be analyzed in subsequent links.

### Model estimation

**FIGURE 1:**
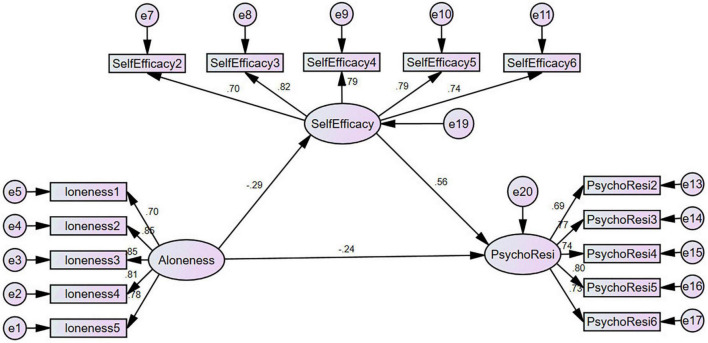
Structure equation modeling results.

According to the path analysis in Table 3, loneliness has a significantly negative impact on SelfEfficacy, β = -0.292, *P* < 0.001; SelfEfficacy had a significant positive effect on PsychoResi, β = 0.556, *P* < 0.001; loneliness had a significant negative influence on PsychoResi, β = -0.244, *P* < 0.01. So the final model is as follows (Figure 1).

**TABLE 3:** Path analysis results.

Path	Standardized path coefficient	Non-standardized path coefficient	Standard error	Critical ratio	Significance
			
			S.E.	C.R.	*P*
SelfEfficacy <— Loneliness	-0.292	-0.227	0.063	-3.597	***
PsychoResi <— SelfEfficacy	0.556	0.44	0.072	6.129	***
PsychoResi <— Loneliness	-0.244	-0.151	0.044	-3.397	***

***p < 0.001.

### Intermediate effect test

This study uses the Bootstrap method of AMOS16.0 software to test the intermediate effect. This method can estimate the direct effect (the effect of the independent variable on the dependent variable after controlling the intermediate variable), the indirect effect (the effect of the intermediate variable on the dependent variable after controlling the independent variable), and the total effect. Data analysis selects 5,000 repeated samples of Bootstrap to determine whether 0 is included in the 95% confidence interval to check whether the mediation effect exists.

The final result is that the effective value of the total effect is -0.278; the effect value of the direct effect test is -0.111, accounting for 39.93%; the effect value of the indirect effect test is -0.167, with an effect accounting for 60.07%. Specifically, SelfEfficacy has a significant mediation in loneliness and PsychoResi, with effect values of -0.111 and *p* < 0.01. Using the Bias-corrected method, it is shown as [-0.198, -0.042] under the condition of 95% confidence interval calculation, and using the Percentile method, it is shown as [-0.191, -0.038]. The confidence interval does not contain 0, and the intermediate effect accounts for 39.93%.

## Discussion

COVID-19 is still spreading all over the world, and its high infectivity has attracted the attention of all countries. The World Health Organization has also listed it as a public health emergency of international concern (Alhazzani et al., 2020). This has brought a lot of uncertainty to the future study and life of college students, to some extent making them confused and doubtful about whether they can effectively control the impact of the epidemic on their life planning. Self-efficacy is a belief that refers to an individual’s ability to control events that affect one’s life (Benight and Bandura, 2004). This study found that although COVID-19 has an impact on students’ health, college students have improved the level of problem-solving and self-efficacy in the process of constantly obtaining new knowledge, which enables them to face the health threat caused by the epidemic in a more positive and rational attitude. Therefore, this also shows that the improvement of self-efficacy plays a great role in psychological resilience, which is consistent with the research results of Ye et al. (2016), that is, the higher the level of self-efficacy of college students, the stronger the ability to control the impact of events when they encounter trauma, and the more they grow after the trauma. Relevant studies have also reported that young people are more likely to change their original thinking, cognition, world outlook, etc. in crisis events to adapt to changes in real life, which helps them achieve post-traumatic growth (Liu et al., 2017). College students are in the later stage of youth, a special period of life development. From the perspective of developmental psychology, this period is a critical period for the formation and establishment of self-identity. Erikson and Erikson (1981) believe that self-identity is a multi-level and multi-dimensional psychological concept closely related to the development of self and personality, which essentially refers to the continuity, maturity, and integration of personality development. It has an important impact on the individual’s social adaptation and mental health. The better the development of College Students’ self-identity, the better the social adaptation. As the survey object of this study is only for entrepreneurial college students, there are certain limitations, so the research conclusions need to be further demonstrated and analyzed. There is still room for improvement in future research. In the future, we will continue to systematically track and investigate the psychological resilience of the students in China’s colleges and universities, find out the gap, and carry out targeted management.

## Conclusion

To sum up, based on the level of psychological resilience, this paper makes a statistical analysis of the mental health of College Students in the background of the COVID-19 epidemic. The results show that self-efficacy is the mediator between loneliness and psychological resilience in targeted Chinese students. Here are some suggestions for future pedagogy:

(1) In school education, targeted educational methods should be adopted according to the emotional characteristics of college students in different grades. For first-year students, public courses in psychology can be offered to them to learn how to maintain mental health. For senior students, because they’re facing the critical moment to enter society, schoolteachers should offer relevant courses on career planning catering to their needs, so that they can positively deal with the situation.
(2) A variety of activities should be held to engage students to provide them with more opportunities for exercise and learning. Students can have a deeper understanding of themselves, keep in closer contact with others, and enhance the relationship between them to reduce troubles through activities with the theme of college students’ mental health knowledge such as self-awareness and emotional regulation skills, and through educational and entertaining activities such as games and knowledge competitions, etc.

This study attempts to fill the gap in research on the psychological status of post- epidemic college students and is conducive to providing a reference for the analysis of the epidemic based on the research of college students in this paper. Due to the lack of references from previous studies, the discussion of the results in this study appears preliminary. With the increase of relevant studies in the future, the discussion of the research results can be further enriched.

## Data availability statement

The raw data supporting the conclusions of this article will be made available by the authors, without undue reservation.

## Author contributions

HG, YZ, and WZ performed the material preparation, data collection, and analysis. HG and WZ wrote the first draft of the manuscript. YT and LY commented on previous versions of the manuscript. All authors contributed to the study conception and design, read, and approved the final manuscript.
